# Duplicated Vermiform Appendix (Type B2) in Complicated Acute Appendicitis: A Pediatric Case Report

**DOI:** 10.7759/cureus.91981

**Published:** 2025-09-10

**Authors:** Guillermo Lawers Cuen, Alexia Y Martínez Gómez, Salvador Pelayo González, Lizbeth Ramirez Sanchez, Pedro I Aquino Solís, Quitzia L Torres Salazar

**Affiliations:** 1 General Surgery, Hospital General de León, León, MEX; 2 Biomedical Sciences, Universidad Juárez del Estado de Durango, Durango, MEX

**Keywords:** appendiceal duplication, complicated appendicitis, congenital gastrointestinal malformation, pouchet’s technique, type b2 anomaly

## Abstract

Appendiceal duplication is a rare congenital anomaly with important diagnostic and surgical implications, particularly when it remains undetected during appendectomy. We present the case of a 16-year-old male with an eight-day history of progressive abdominal pain that evolved into generalized peritonitis. Laboratory evaluation revealed leukocytosis (25,530/mm³), neutrophilia (83%), and elevated C-reactive protein (277.2 mg/L). Abdominal ultrasound demonstrated findings consistent with complicated appendicitis. Exploratory laparotomy identified two gangrenous appendices with independent bases and mesoappendices, corresponding to type B2 duplication. Both structures were resected using Pouchet’s technique. Histopathological examination confirmed acute suppurative, necro-hemorrhagic, perforated appendicitis in both specimens. The postoperative course was favorable, and the patient was discharged on the second postoperative day. This case highlights the clinical relevance of meticulous intraoperative inspection of the cecum and retrocecal space to detect unusual anatomical variants, ensuring complete surgical management and preventing recurrence or postoperative complications.

## Introduction

Appendiceal duplication is an extremely rare congenital anomaly, with an estimated incidence of 0.004% to 0.009% [1]. Although most anomalies of the appendix are clinically silent, duplication has important surgical and medicolegal implications, particularly when one appendix remains undetected during appendectomy. Failure to identify and remove both appendices may result in persistent or recurrent symptoms, leading to reoperations and potential legal consequences [2,3].

The most widely accepted classification system is the Cave-Wallbridge classification, initially described by Cave in 1936 and revised by Wallbridge in 1963, which categorizes duplications into four types (A-D) according to anatomical configuration. Type B2, in which one appendix arises from the usual location and a second arises along the tenia coli, is the most frequently reported form and is typically discovered incidentally during surgery [4].

Because preoperative imaging rarely detects double appendices, diagnosis is generally made intraoperatively [1]. This case highlights the clinical and surgical relevance of appendiceal duplication, emphasizing the importance of careful cecal inspection during appendectomy.

This report aims to present a pediatric patient with complicated acute appendicitis in the context of appendiceal duplication (type B2) and to discuss its implications in light of current literature. This case has been reported in accordance with the SCARE (Surgical Case Report) 2025 guidelines [5].

## Case presentation

A 16-year-old male with no relevant past medical or surgical history presented with an eight-day history of abdominal pain initially localized to the hypogastrium (8/10 intensity) and associated with anorexia and vomiting. He self-medicated with butylscopolamine, obtaining partial relief. The pain later migrated to the right iliac fossa. He was initially evaluated by a primary care physician, diagnosed with a urinary tract infection, and treated with phenazopyridine and trimethoprim-sulfamethoxazole without improvement over the following week. Subsequently, he developed fever and diarrhea, prompting presentation to the emergency department of the General Hospital of León with severe, generalized abdominal pain (10/10).

On admission, vital signs were temperature 38°C, heart rate 98 bpm, blood pressure 124/74 mmHg, respiratory rate 18 breaths/min, and oxygen saturation 96%. Physical examination revealed an alert, oriented patient with mildly dry oral mucosa. Abdominal examination demonstrated marked tenderness and rebound in the right iliac fossa, with positive McBurney’s, Rovsing’s, and rebound signs.

**Figure 1:**
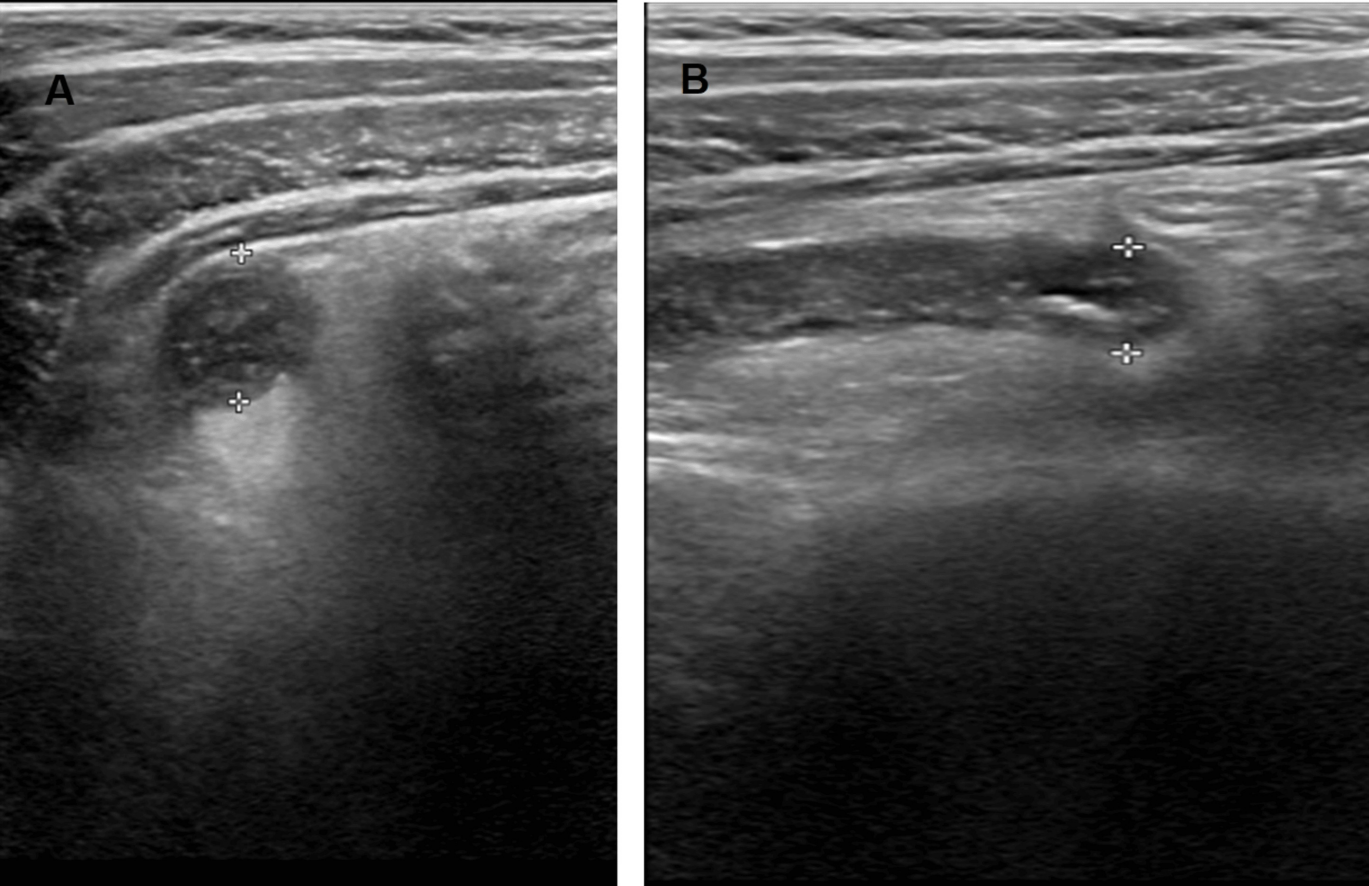
Abdominal ultrasound showing the cecal appendix with increased diameter, appendiceal wall thickening, and the presence of periappendiceal fluid (A) Transverse view demonstrating a distended appendiceal lumen with hypoechoic wall thickening. (B) Longitudinal view revealing diffuse mural thickening and adjacent fluid.

Laboratory investigations showed leukocytosis (25,530/mm³) with neutrophilia (83%) and elevated C-reactive protein (277.2 mg/L) (Table 1). Abdominal ultrasound revealed free fluid in the pelvic cavity, increased echogenicity of pericecal fat, and mesenteric lymphadenopathy (Figure 1). The appendix appeared aperistaltic with a wall thickness of 3.9 mm. A diagnosis of complicated acute appendicitis was made, and the patient was scheduled for emergency surgery.

**Table 1:** Laboratory results at admission showed marked leukocytosis with neutrophilia and elevated C-reactive protein, consistent with complicated acute appendicitis

Parameter	Value	Reference range
Leukocytes	25,530/mm³	4,000-10,000/mm³
Neutrophils	83%	40-70%
C-reactive protein	277.2 mg/L	<5 mg/L
Hemoglobin	13.5 g/dL	13-17 g/dL
Platelets	320 ×10³/µL	150-400 ×10³/µL
Glucose	92 mg/dL	70-100 mg/dL
Creatinine	0.8 mg/dL	0.6-1.3 mg/dL

**Figure 2:**
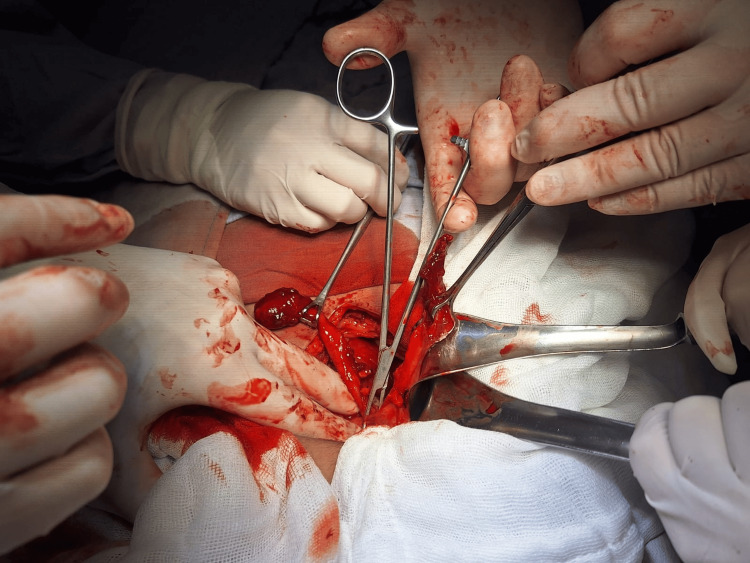
Intraoperative view showing type B2 appendiceal duplication. Both appendices are visualized with independent bases and mesoappendices during dissection of the cecal pole The surgical field demonstrates the separation of inflammatory adhesions to allow complete resection.

A midline infraumbilical laparotomy was performed, revealing approximately 100 mL of purulent fluid in the peritoneal cavity and an inflammatory mass involving the terminal ileum, sigmoid colon, cecum, and urinary bladder. Dissection of the phlegmon revealed a cecal appendix with a preserved base, gangrenous changes, and a perforated tip, adherent to a second tubular structure parallel to it, also exhibiting tip necrosis. Further dissection revealed that the second structure had its own independent base in the cecum. Both appendices had separate mesoappendices and independent vascular pedicles, consistent with type B2 appendiceal duplication. Both were in a gangrenous phase with tip necrosis and pelvic adhesions (Figure 2).

**Figure 3:**
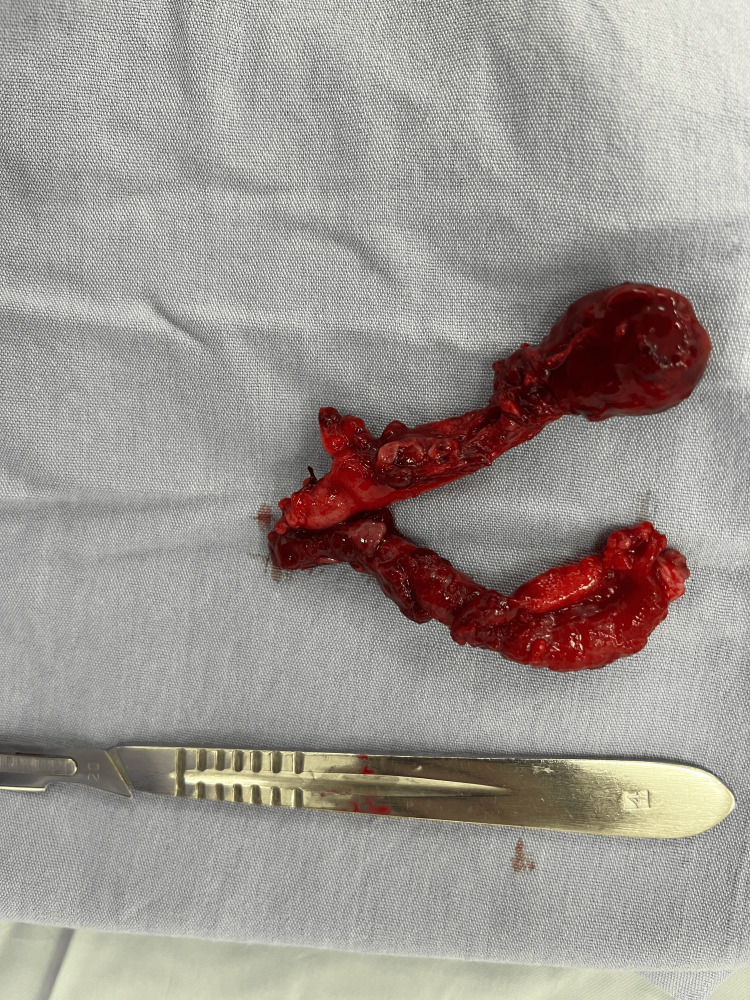
Gross surgical specimen showing two independent vermiform appendices, each with its own mesoappendix and vascular pedicle, consistent with type B2 appendiceal duplication Both appendices exhibit gangrenous changes, with necrosis at the tip.

**Figure 4:**
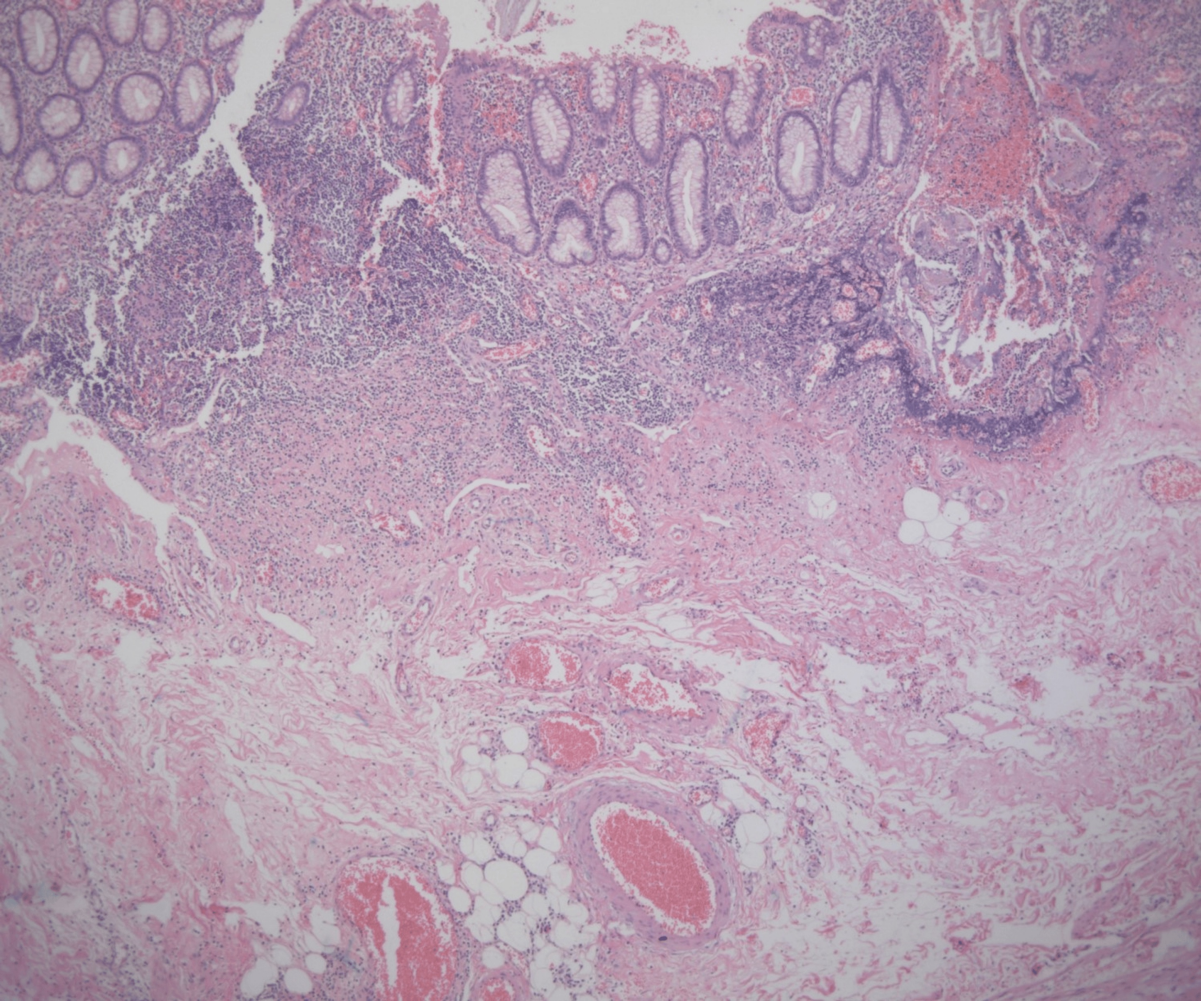
Low-power photomicrograph (H&E, 5×) of the appendix demonstrating mucosa lined by simple columnar epithelium with focal erosions There is a prominent acute inflammatory infiltrate composed of neutrophils, lymphocytes, and plasma cells, extending transmurally to involve the serosa. Foci of mural discontinuity are present, consistent with perforation. H&E: hematoxylin and eosin

A double appendectomy was performed using Pouchet’s technique. In this approach, the cecum is exposed, and the free tenia coli is followed to its convergence, where the appendiceal base is usually located. Once the appendix is identified, the mesoappendix is dissected, and the appendiceal artery is ligated and divided. The appendix is then clamped approximately 1 cm above the base, transected, and the stump ligated with 2-0 silk without invagination, provided the base is intact and not necrotic or perforated. In our case, simple ligation of each base was performed using 2-0 silk. The abdominal wall was closed in layers with 1-0 Vicryl for fascia and interrupted 3-0 nylon sutures for skin. Both specimens were sent to pathology as “Appendix 1” and “Appendix 2" (Figure 3). Gross examination described two cecal appendices measuring 7 × 2 × 1 cm and 6 × 1.5 × 1 cm, respectively, each with yellowish serosa, purulent exudate, necrotic areas, and wall discontinuity. Histopathological analysis confirmed acute suppurative, necro-hemorrhagic, and perforated appendicitis with associated acute periappendicitis. No evidence of malignancy was identified (Figure 4).

The postoperative course was uneventful. The patient received broad-spectrum intravenous antibiotics, tolerated diet by postoperative day 2, and was discharged home after 48 hours in stable condition. Follow-up was conducted at 10 days for suture removal and subsequently at three months in the general surgery outpatient clinic. The patient remained asymptomatic, tolerated oral intake, and reported normal bowel movements without complications.

## Discussion

Appendiceal duplication, while uncommon, is a congenital anomaly of surgical relevance due to its diagnostic difficulty and potential consequences if unrecognized. Most duplications are diagnosed intraoperatively, as preoperative imaging (whether ultrasonography or computed tomography) rarely identifies the anomaly, particularly in cases of type B2 appendiceal duplication, where the second appendix may be retrocecal or embedded along the tenia coli. In our patient, as in the cases described by Christodoulidis et al. and Ayoub et al., ultrasonography confirmed inflammatory changes but failed to identify the duplication [6,7].

According to the Jerusalem guidelines of the World Society of Emergency Surgery (2020), the use of imaging in acute appendicitis should be tailored to the pretest probability of the disease. Ultrasonography is recommended as the first-line study when imaging is required due to diagnostic uncertainty. At the same time, contrast-enhanced CT is reserved for cases of negative or inconclusive ultrasound or persisting doubt [8]. In our case, the patient presented with a typical clinical picture of acute appendicitis with high preoperative probability, and ultrasonography revealed findings consistent with complicated appendicitis; therefore, the decision was made to proceed with open appendectomy without CT. Moreover, appendiceal duplication is usually an intraoperative finding, and CT would not have altered the therapeutic approach in this scenario. In terms of surgical management, the literature consistently recommends resection of both appendices once duplication is identified, even if one appears macroscopically normal. This is because the duplicated mucosa retains the potential for future inflammation, ulceration, or perforation, which could lead to recurrent appendicitis.

Although the cecal appendix has well-established immunological functions, including maintaining intestinal microbiome homeostasis and acting as mucosa-associated lymphoid tissue, there is no evidence to suggest that preserving an accessory appendix provides any significant clinical benefit. On the contrary, the potential risks of recurrent appendicitis or even neoplastic transformation justify resection. In the present case, both appendices showed macroscopic evidence of inflammation and were therefore removed.

The Cave-Wallbridge classification remains the standard for categorizing appendiceal duplications. Type B2, as in our case, is considered the most frequent variant. In this subtype, one appendix originates from the normal anatomical location, while the second arises separately from the cecum along the tenia coli. This positioning increases the likelihood of overlooking the second appendix during standard exploration, primarily if the surgeon’s attention is focused on an obviously inflamed structure [4].

The clinical presentation of appendiceal duplication varies. While most patients present with inflammation of only one appendix, bilateral appendicitis is rare. In the review cited by Ayoub et al., fewer than 15 cases involved concurrent inflammation of both appendices [7]. Our case contributes to the limited number of reports of bilateral advanced appendicitis, which has also been documented by Markou et al. and Alsaud et al. [1,3]. The bilateral gangrenous changes with perforation observed in our patient underscore the potential for severe intra-abdominal sepsis in undiagnosed duplications.

From a surgical perspective, intraoperative identification of appendiceal duplication requires a systematic and meticulous approach. Multiple authors have emphasized the need to examine the entire cecal circumference, retrocecal space, and the course of the tenia coli to detect a second appendix. Markou et al. emphasize that in type B2 appendiceal duplication, the second appendix often lies posteriorly and may escape detection unless specifically sought [1]. Christodoulidis et al. highlight the medicolegal implications of missed duplications, as recurrent abdominal pain, stump appendicitis, or future complications may arise, potentially leading to litigation [6]. Regarding the routine exposure of the appendiceal lumen for duplication screening, the only potential benefit would be the confirmation of unusual luminal configurations or anatomical variants. However, this approach has significant drawbacks, including prolongation of surgical time and an increased risk of contamination. There is no evidence supporting its routine use, given the extreme rarity of appendiceal duplication. The standard technique emphasizes identifying the cecum and teniae to locate the base of the appendix. In our case, the appendiceal lumen was not opened; nevertheless, because Pouchet’s technique leaves both appendiceal stumps uninverted, the mucosa of each stump arising from the cecum could be directly visualized.

Histopathological confirmation of both specimens is essential to validate the diagnosis. In our case, the independent anatomical bases and separate mesoappendices were corroborated histologically, aligning with the diagnostic criteria outlined by Handra-Luca et al. This step is crucial in cases with complicated appendicitis, where inflammatory changes can obscure anatomical details intraoperatively.

The pathogenesis of appendiceal duplication remains incompletely understood. Cave proposed two theories: persistence of a transient embryologic structure or a localized duplication as part of a broader midgut developmental anomaly. Although types B1 and C are more often associated with other congenital anomalies of the gastrointestinal or genitourinary tract, our case, consistent with other B2 reports, presented without additional malformations.

## Conclusions

Type B2 appendiceal duplication, although rare, is the most frequently encountered variant and carries a significant risk of being overlooked during appendectomy. Complicated appendicitis may involve both appendices and lead to severe intra-abdominal sepsis if unrecognized. Careful intraoperative inspection of the cecal pole, retrocecal space, and tenia coli is essential to identify a second appendix. Complete resection of both appendices, with histopathological confirmation, is critical to prevent recurrence and to avoid potential medicolegal consequences.
